# Carbon Dots from Seaweed Biomass: Characteristics, Bioactivities and Retardation of Lipid Oxidation in Pacific White Shrimp During Refrigerated Storage

**DOI:** 10.3390/foods15152636

**Published:** 2026-07-27

**Authors:** Harisankar Kunnamkulathil Chandrababu, Gokulprasanth Murugan, Suriya Palamae, Jirakrit Saetang, Prabjeet Singh, Rangasamy Anandan, Yadong Zhao, Bin Zhang, Yu Fu, Soottawat Benjakul

**Affiliations:** 1 International Center of Excellence in Seafood Science and Innovation (ICE-SSI), Faculty of Agro-Industry, Prince of Songkla University, Hat Yai 90110, Songkhla, Thailand; hskc03@gmail.com (H.K.C.); gokulprasanth031999@gmail.com (G.M.); suriya.pal@psu.ac.th (S.P.); jirakrit.s@psu.ac.th (J.S.); 2 Department of Food Technology, School of Bio, Chemical and Processing Engineering (SBCE), Kalasalingam Academy of Research and Education (KARE), Anand Nagar, Krishnankoil, Virudhunagar 626126, Tamil Nadu, India; 3 Farm Science Centre (Tarn Taran), Guru Angad Dev Veterinary and Animal Sciences University, Ludhiana 141004, Punjab, India; prabjeet29255@yahoo.co.in; 4 Biochemistry and Nutrition Division, ICAR-Central Institute of Fisheries Technology, Cochin 682029, Kerala, India; kranandan@rediffmail.com; 5 Key Laboratory of Health Risk Factors for Seafood of Zhejiang Province, College of Food Science and Pharmacy, Zhejiang Ocean University, Zhoushan 316022, China; zhaoyd@zjou.edu.cn (Y.Z.); zhangbin@zjou.edu.cn (B.Z.); 6 College of Food Science, Southwest University, No.2. Tiansheng Road, Beibei District, Chongqing 400715, China; fuy987@swu.edu.cn; 7 Department of Food and Nutrition, Kyung Hee University, 26 Kyungheedae-ro, Dongdaemun-gu, Seoul 02447, Republic of Korea

**Keywords:** seaweed-derived carbon dots, antioxidant, anti-microbial, hydrothermal process, SDG 12: Responsible consumption and production

## Abstract

Seaweeds are valuable marine resources rich in polysaccharides and polyphenols. Carbon dots (CDs) from *Gracilaria salicornia* (GS-CDs), *Halymenia dilatata* (HD-CDs), *Sargasum polycystum* (SP-CDs), *Spatoglossum asperum* (SA-CDs), *Ulva lactuca* (UL-CDs), and *Caulerpa peltata* (CP-CDs) were synthesized by a hydrothermal process and characterized by FTIR, XPS, and SEM-EDX. All the CDs had sizes less than 10 nm with spherical morphology and various functional groups. UL-CDs showed the strongest UV-A blocking efficacy via the measurement of light transmission (*p* < 0.05). HD-CDs exhibited the highest DPPH-RS-A (148.32 ± 1.51 μmol TE/L) and FRA-P (717.24 ± 7.87 μmol TE/L), whereas SA-CDs had the highest ABTS-RS-A (1043.40 ± 3.00 μmol TE/L). HD-CDs had antifungal activity against both *Aspergillus flavus* and *Aspergillus parasiticus*. All CDs suppressed the proliferation of both pathogenic and spoilage bacteria; however, high MIC values indicated the limited antibacterial effectiveness. CDs up to 500 mg/L maintained cell viability greater than 80% towards normal BJ cells. When SA-CDs (500 ppm) were incorporated into peeled and deveined Pacific white shrimp (*Litopenaeus vannamei*), lipid peroxidation during 10 days of refrigerated storage was retarded, as evidenced by lower PV, TBARS, and greater PUFA retention than the control and ascorbic acid-treated samples. Heat map and PCA analyses revealed that lipid oxidation was governed by CD type and storage time.

## 1. Introduction

Seaweed, commonly referred to as marine macroalgae, is a diverse group of photosynthetic organisms that inhabit coastal and marine ecosystems. They are broadly classified into three major groups based on pigmentation and biochemical characteristics: brown algae (Phaeophyceae), red algae (Rhodophyceae), and green algae (Chlorophyceae) [1]. These organisms serve as primary producers in marine environments and contribute significantly to ecological balance and nutrient cycling. In addition to their ecological role, seaweed has attracted growing attention due to a rich array of beneficial chemical components [2]. Marine macroalgae are known to contain a wide array of bioactive compounds, including polysaccharides such as alginate, carrageenan, and agar, along with polyphenols, pigments, proteins, vitamins, and essential minerals [3]. These compounds exhibit various biological activities, including antioxidant, antimicrobial, anti-inflammatory, antiviral, and anticancer properties [4]. The synthesis of such metabolites is often connected to the adaptive mechanisms of seaweeds to fluctuating and harsh environmental conditions [5]. The uncontrolled proliferation of seaweeds blocks sunlight from reaching the water column and depletes dissolved oxygen, leading to the degradation of coral reefs, seagrass meadows, fisheries, and overall marine biodiversity [6]. As the stranded biomass decomposes along the shoreline, it releases foul-smelling gases such as hydrogen sulfide and ammonia, posing health risks to nearby communities, discouraging tourism, and imposing substantial clean-up costs on coastal regions [7]. Transitioning this problematic waste biomass into high-value functional materials has therefore emerged as a compelling strategy within the circular bioeconomy. The green synthesis of seaweed-derived CDs (SWCDs) supports SDG 12 through biomass valorization, SDG 14 by promoting sustainable marine resource use, and SDGs 9 and 13 through sustainable nanotechnology and low-carbon material development.

Nanomaterials including metal nanoparticles such as silver and zinc oxide, nano emulsions and polymer-based nanocomposites, have been explored as additives or packaging material to enhance food quality and safety [8]. In recent years, carbon dots (CDs) have emerged as a novel class of zero-dimensional carbon-based nanostructures with particle sizes typically below 10 nm [9]. CDs possess unique physicochemical properties, including strong photoluminescence, high water solubility, chemical stability, low toxicity, and tunable surface functionalities. These characteristics enable their application in diverse fields such as bioimaging, sensing, catalysis, and drug delivery [10].

Green synthesis of CDs by the hydrothermal process using natural precursors has gained increasing attention since it offers several advantages such as environmental sustainability, cost-effectiveness, and the avoidance of toxic reagents [11]. CDs from various natural precursors like tangerine peel, cashew, and jik leaves were synthesized using the hydrothermal method [12,13]. During the hydrothermal process, biomass precursors undergo hydrolysis, dehydration, polymerization, aromatization, and carbonization under elevated temperature and pressure, leading to the formation of nanosized carbon cores with surface passivation [14]. Seaweeds have emerged as promising candidates for CDs synthesis due to their abundance and high contents of polysaccharides, amino acids, and phenolic compounds [15]. These biomolecules can be converted to CDs with enhanced surface functional groups such as hydroxyl, carboxyl, amino, and sulfate groups, thus enhancing the bioactivities, particularly antioxidant and antimicrobial properties [16]. CDs have been used in food systems such as food quality monitoring, active packaging, and the development of functional additives [17]. Their inherent antioxidant and antimicrobial properties make them suitable for improving food stability and safety [18]. However, most studies have focused on the incorporation of CDs into film-based active packaging systems, while their direct application of seaweed-derived CDs in food matrices is still limited [19].

Although natural preservatives such as essential oils, plant extracts, and polyphenols have shown promising antioxidant and anti-microbial activities, their direct application is often limited by poor stability and strong sensory impacts [20,21]. Recently, various nanomaterials, such as CDs derived from several biomass or agricultural wastes, such as fruit peel, leaves, legume seed coat, stick water, etc., have emerged as a promising alternative to conventional synthetic antioxidants due to their excellent biocompatibility, ultra-small size, and higher water solubility [22]. Shrimp are among the most commercially important crustaceans, having high nutritional content and desirable taste. They are rich in high-quality proteins, essential amino acids, and polyunsaturated fatty acids like EPA and DHA [23,24]. Even though the lipid content of shrimp is low, at 0.7–1.2% (*w*/*w*), lipid oxidation in the muscles of shrimp will decrease the nutritional value and negatively affect the sensory quality [24]. Therefore, CDs with high antioxidant activity can conquer such a problem. However, CDs from a variety of seaweeds might serve as promising antioxidants for shrimp treatment. Nonetheless, little information on the bioactivities of CDs from different types of seaweed exists. Accordingly, the present study aimed to synthesize CDs from different seaweed species using a hydrothermal method and systematically characterize their physicochemical properties and bioactivities. Furthermore, the synthesized SWCDs were directly applied to peeled and deveined Pacific white shrimp to evaluate their effectiveness in suppressing lipid oxidation during refrigerated storage. Thus, the study provides a foundation for developing sustainable, seaweed-derived nanomaterials as natural antioxidant additives or alternative preservatives for seafood preservation.

## 2. Materials and Methods

### 2.1. Chemicals

The chemicals used in this investigation were of analytical grade, and the reagents were purchased from Sigma-Aldrich (St. Louis, MO, USA). The microbiological media, including Mueller–Hinton Agar (MHA), Mueller–Hinton Broth (MHB), plate count agar (PCA), tryptic soy agar (TSA), and tryptic soy broth (TSB), were acquired from HiMedia Laboratories Pvt. Ltd. (Mumbai, India).

*Shewanella putrefaciens* (JCM 20190) was obtained from the Japan Collection of Microorganisms (JCM), RIKEN BioResource Research Center (Ibaraki, Japan). *Escherichia coli* (ATCC 25922), *Staphylococcus aureus* (ATCC 25923), and *Pseudomonas aeruginosa* (ATCC 27853) were obtained from the American Type Culture Collection (ATCC, Manassas, VA, USA). *Listeria monocytogenes* (FSL J1-208) was obtained from the Food Safety Laboratory (FSL), Cornell University (Ithaca, NY, USA). The fungal strains, namely *Aspergillus flavus* (PSRDC-4) and *Aspergillus parasiticus* (TISTR 3276), were obtained from the Thailand Institute of Scientific and Technological Research (TISTR, Khlong Luang, Pathum Thani, Thailand). Human BJ fibroblasts (ATCC CRL-2522), fibroblast basal medium (FBM), and fibroblast supplemental growth factors were acquired from the American Type Culture Collection (ATCC, Manassas, VA, USA).

### 2.2. Green Synthesis of Carbon Dots (CDs) from Different Seaweeds

Different seaweeds were collected from a private farm (R. K. Algae Project Centre) located in the Mandapam coastal region on the south-east coast of Tamil Nadu, India. The collected seaweeds comprised red seaweed (*Halymenia dilatata* and *Gracilaria salicornia*), green seaweed (*Ulva lactuca* and *Caulerpa peltata*), and brown seaweed (*Sargassum polycystum* and *Spatoglossum asperum*). The obtained samples were washed and dried at 50 °C using an electric dryer (Kraftwork drier-KSD 100, Kraftwork solar Pvt Ltd, Kochi, India) for 72 h. The dried samples with moisture contents of 3% were powdered using a household blender and packed in polythene bags, heat-sealed and stored at 4 °C until further use.

A suspension of seaweed powder (2%) in distilled water was stirred for 10 min, placed in a Teflon-lined cylinder, sealed in a stainless-steel reactor, and subsequently heated in a muffle furnace (200 °C, 6 h) [12]. The yellow–brownish solution obtained was then filtered using a Whatman filter paper (cut off: 100 µm) and the filtrate was further filtered using a syringe filter (pore size: 0.22 µm) and labeled as *Halymenia dilatata* CDs (HD-CDs), *Gracilaria salicornia* CDs (GS-CDs), *Ulva lactuca* CDs (UL-CDs), *Caulerpa peltata* CDs (CP-CDs), *Sargassum polycystum* CDs (SP-CDs) and *Spatoglossum asperum* CDs (SA-CDs) and stored at 4 °C for further analysis.

### 2.3. Characterization of CDs Synthesized from Different Seaweeds

#### 2.3.1. Analyses of Visual Appearance, Transmission Electron Microscopic (TEM) Images, Scanning Electron Microscopy–Energy-Dispersive X-Ray (SEM-EDX) Spectra, and Yield

The visual appearance of SWCDs under normal light was captured by a smartphone, whereas their appearance under UV light exposure was captured by a UVITEC Cambridge gel documentation system. The morphology of the synthesized SWCDs was characterized using a field-emission transmission electron microscope (FE-TEM) (Talos F200i, Thermo Scientific Co. Ltd., Waltham, MA, USA) [12]. The elemental composition of the dried SWCDs was analyzed using a scanning electron microscope equipped with an energy-dispersive X-ray spectroscopy detector (Hitachi SU3900, Hitachi High-Tech Corporation, Tokyo, Japan). [25].

To determine the yield of CDs, the filtrate containing CDs was dried in a hot air oven at 105 °C for 18–24 h, and the obtained solids were weighed. The percentage yield was then calculated relative to the dry weight of seaweed powder used as the precursor [26].

#### 2.3.2. UV–Vis Spectrophotometric and Spectrofluorometric Spectra Analysis

The UV–visible absorption spectra of the SWCDs were recorded using a UV–Vis spectrophotometer (Shimadzu, Model UV-1800, Kyoto, Japan) over a wavelength of 200–800 nm. A solution at 50 ppm of each SWCD was used for analysis [27]. Fluorescence spectral measurement of SWCD solution was done using a spectrofluorometer (Hitachi, Model F-7100 FL, Tokyo, Japan) at 25 °C. The SWCDs diluted with distilled water were analyzed over an excitation wavelength of 200–400 nm, and the corresponding emission spectra were collected at 300–550 nm with an increment of 10 nm. The slit width and scan speed were set at 5 nm and 1200 nm/min, respectively [27].

#### 2.3.3. Fourier Transform Infrared (FTIR) Spectra and X-Ray Photoelectron Spectroscopic (XPS) Analysis

FTIR spectra of SWCDs (freeze-dried powder) were recorded using a Bruker FTIR spectrometer (Model Equinox 55, Bruker Co., Ettlingen, Germany). The analysis was performed over a wavenumber range of 4000–400 cm^−1^ for the identification of functional groups and secondary structures, in which 32 scans and 4 cm^−1^ resolution were used. Samples were mixed with KBr at a ratio of 1:10 using a mortar and pestle and compressed into pellets before analysis. OPUS 3.0 data collection software was utilized to normalize the spectra prior to interpretation [27].

X-ray photoelectron spectroscopy (XPS; AXIS ULTRADLD, Kratos Analytical, Manchester, UK) was used to determine the elemental composition and surface chemical bonding states of synthesized CDs [28].

#### 2.3.4. Determination of UV-Blocking Property

The UV barrier property was examined, and the blocking ability toward UV-A and UV-B was computed using the following Equations (1) and (2):

(1) $$UV-A\;blocking\left(\%\right)=100-\frac{\int_{320}^{400}T\left(\lambda \right)d\lambda }{\int_{320}^{400}d\lambda }$$

(2) $$UV-B\;blocking\left(\%\right)=100-\frac{\int_{280}^{320}T\left(\lambda \right)d\lambda }{\int_{280}^{320}d\lambda }$$

where *T*(*λ*) is the average transmittance of SWCDs at the wavelength *λ*, and *dλ* is the bandwidth interval [29].

### 2.4. Evaluation of Antioxidant, Antimicrobial Activities and Cytotoxicity 

#### 2.4.1. Antioxidant Activities

To evaluate the antioxidant and metal chelating activity of the synthesized SWCDs, four distinct spectrophotometric assays were performed. For the DPPH radical scavenging activity (DPPH-RS-A), 500 µL of sample was mixed with an equal volume of 0.15 mM DPPH solution, and the absorbance was measured at 517 nm after a 30 min incubation. For the ABTS radical scavenging activity (ABTS-RS-A), the stock solution includes 7.4 mM ABTS and 2.6 mM potassium persulfate. The working solution was prepared by mixing the two stock solutions in equal quantities and allowing them to react for 12 h at room temperature in the dark. The solution was then diluted by mixing 1 mL of ABTS solution with 50 mL of methanol in order to obtain an absorbance of 1.1 ± 0.02 at 734 nm. A volume of 10 µL of sample with 190 µL working solution was incubated for 45 min, and absorbance was read at 734 nm. A sample blank was prepared by replacing the ABTS solution with methanol. For the FRAP assay, stock solutions including 300 mM acetate buffer (pH 3.6), 10 mM TPTZ (2,4,6-tripyridyl-s-triazine) solution in 40 mM HCl, and 20 mM FeCl_3_·6H_2_O solution were prepared. A working solution was freshly prepared by mixing 25 mL of acetate buffer, 2.5 mL of TPTZ solution, and 2.5 mL of FeCl_3_·6H_2_O solution. The mixed solution was incubated at 37 °C for 30 min in a water bath (Memmert, D-91126, Schwabach, Germany) and was referred to as the FRAP solution. The mixture of 10 µL of sample and 190 µL of FRAP solution was incubated for 30 min at room temperature in the dark, and absorbance was read at 593 nm. A sample blank was prepared by replacing FeCl_3_ with distilled water in the FRAP solution. To determine metal chelating activity (MC-A), a 940 µL aliquot of sample was sequentially treated with 20 µL of 2 mM ferrous chloride and 40 µL of 5 mM ferrozine, followed by incubation for 30 min. Finally, the absorbance was read at 562 nm. Final values for DPPH-RS-A, ABTS-RS-A, and FRA-P were calculated and expressed as µmol Trolox equivalent (TE)/L sample. MC-A was computed and reported as µmol EDTA equivalent (EE)/L sample [30].

#### 2.4.2. Antimicrobial Activity

Antifungal activities of all SWCDs at various levels (3–10 mg/mL) against *Aspergillus flavus* (AF) and *Aspergillus parasiticus* (AP) were tested in comparison with the control (distilled water) following the method of Murugan et al. [12]. The growth of fungi was expressed in diameter (mm).

The MIC values of SWCDs against both pathogenic bacteria (*Listeria monocytogenes*, *Staphylococcus aureus*, *Escherichia coli*) and spoilage bacteria (*Shewanella putrefaciens*, *Pseudomonas aeruginosa*) were determined by resazurin microdilution assay [29]. Bacterial cultures were prepared and inoculated into tryptic soy broth (TSB) and incubated in a shaking incubator at 37 °C and 180 rpm for 3 h. The bacterial turbidity was initially adjusted to be equivalent to 0.5 McFarland standard. Subsequently, the broth was further diluted to attain a final concentration of 0.5 × 10^6^ CFU/mL. The samples were serially diluted (125–0.24 mg/mL) using MHB in a sterile 96-well plate. To each well, 100 µL of bacterial suspension was added, and the plates were incubated at 37 °C for 24 h. After the incubation, resazurin solution (20 µL) was added and further incubated for 4 h. The MIC was defined as the lowest concentration that prevented the color change in the indicator from purple to pink. For the determination of MBC, 10 µL of aliquots from wells without visible bacterial growth were plated on tryptic soy agar (TSA) and incubated at 37 °C for 24 h. The lowest concentration at which there was no bacterial colony formation in agar plates was recorded as the MBC.

#### 2.4.3. Cytotoxicity

Cytotoxicity of SWCDs was investigated using BJ human fibroblast cells following an MTT cell viability assay [27]. BJ cells were cultured in Eagle’s Minimum Essential Medium (EMEM) in the presence of 10% fetal bovine serum and 1% penicillin–streptomycin solution to ensure growth and to prevent microbial contamination. The cells were cultured in tissue culture flasks and incubated at 37 °C in a humid environment with 5% CO_2_. Culture medium was changed every two to three days, and the cells were subcultured when they attained about 80 to 90 percent confluence using 0.25% trypsin–EDTA for detachment.

Upon reaching confluency, the BJ cells were seeded into 96-well plates at a density of 10,000 cells per well and allowed to grow for 24 h under standard incubation conditions. Following the incubation, the cells were treated with SWCDs at different concentrations (1–125 mg/mL) and incubated for 24 and 48 h. The cells without any treatment served as the control. At the end of treatment, MTT solution was added to each well, and the plates were incubated for 2 h to allow viable cells to convert the yellow tetrazolium salt into purple formazan crystals through mitochondrial enzymatic activity. The culture medium was then carefully removed, and dimethyl sulfoxide (DMSO) was added to dissolve the formed crystals. The absorbance was measured at 570 nm. The cytotoxicity was determined based on the relative decrease in cell survival.

### 2.5. Effect of SWCDs on Prevention of Lipid Oxidation of Pacific White Shrimp Meat During Refrigerated Storage

Fresh Pacific white shrimp were purchased from a local market at Hat Yai, Songkhla, Thailand. The shrimp were thoroughly cleaned using tap water. Thereafter, the shrimp were peeled and deveined. The shrimp samples were divided into 9 experimental groups, each containing 15 g of shrimp meat and packed in a polynylon bag (18 cm × 12 cm). Group 1 (the control) was treated with sterile distilled water (C). Group II was treated with distilled water supplemented with chloramphenicol (100 ppm) (DW + CH) to inhibit microbial growth. Group III was treated with ascorbic acid in combination with chloramphenicol (AA + CH) and served as the positive control. Groups IV–IX (HD-CDs + CH, GS-CDs + CH, UL-CDs + CH, CP-CDs + CH, SP-CDs + CH and SA-CDs + CH) were treated with six different SWCDs at a concentration of 500 ppm along with chloramphenicol (100 ppm). Chloramphenicol was used to prevent microbial growth, which might affect the oxidation of shrimp meat. As a consequence, the antioxidant effect of CDs on shrimp lipid could be elucidated without the interfering effect of any actions from microorganisms. All samples were stored under refrigerated conditions and analyzed at 2-day intervals over a 10-day storage period.

#### 2.5.1. Peroxide Value (PV) and Thiobarbituric Acid Reactive Substances (TBARS) Value

PV was determined using the ferric thiocyanate method following the method of Murugan et al. [13]. Briefly, the sample (2 g) was homogenized with 22 mL of chloroform/methanol (2:1, *v*/*v*) at 13,500 rpm for 2 min. The homogenate was then filtered through Whatman No. 1 filter paper, and 7 mL of the filtrate was transferred to a test tube and mixed with 2 mL of 0.5% (*w*/*v*) sodium chloride solution. After vortexing for 30 s, the mixture was centrifuged at 3000× *g* for 3 min at 4 °C to facilitate phase separation. Subsequently, 3 mL of the lower organic phase was collected and mixed with 25 µL of 30% (*w*/*v*) ammonium thiocyanate and 25 µL of 20 mM ferrous chloride solution. The reaction mixture was left to stand at room temperature for 20 min before the absorbance was measured at 500 nm. A reagent blank was prepared by replacing the ferrous chloride solution with distilled water. Peroxide content was quantified using a calibration curve prepared with cumene hydroperoxide standards (0.5–2.0 ppm), and the results were expressed as mg cumene hydroperoxide per kg of sample.

TBARS value was measured as per the procedure of Murugan et al. [13]. Briefly, 0.5 g of sample was homogenized with 2.5 mL of TBARS reagent containing 0.375% (*w*/*v*) thiobarbituric acid (TBA), 15% (*w*/*v*) trichloroacetic acid (TCA), and 0.25 mM hydrochloric acid (HCl). The homogenate was heated in a boiling water bath (95–100 °C) for 10 min to allow color development, after which it was immediately cooled under running tap water. The mixture was then centrifuged at 3600× *g* for 20 min at 25 °C, and the absorbance of the clear supernatant was recorded at 532 nm. Quantification was performed using a calibration curve prepared with 1,1,3,3-tetramethoxypropane (0–6 ppm), and the results were expressed as mg malondialdehyde (MDA) equivalents per kg of sample.

#### 2.5.2. Fatty Acid Profiles

The fresh sample (day 0), positive control, and CD-treated samples showed the lowest PV and TBARS and were subjected to fatty acid analysis. Firstly, lipids were extracted from ground samples from each sample using the Bligh and Dyer method [31]. The extracted lipids (10 mg) were subjected to transmethylation using 2 M methanolic NaOH, followed by 2 M HCl in methanol to obtain FAMEs [32]. FAMEs were injected into a gas chromatography equipped with a flame ionization detector (Agilent 7890B, Santa Clara, CA, USA) and an Omegawax^TM^ 320 fused silica capillary column (30 m × 0.32 mm × 0.25 µm). The conditions used for the GC analysis were as follows: injection temperature of 250 °C and detector (FID) temperature of 270 °C. The initial column temperature of 80 °C was increased to 220 °C at increments of 4 °C min^−1^ for 40 min and further increased to 240 °C at increments of 5 °C min^−1^.

### 2.6. Principal Component Analysis

Principal component analysis (PCA) biplots together with correlation heatmap analysis were applied to investigate the relationships among antioxidant properties, oxidative deterioration parameters, and storage time. Multivariate statistical analyses were conducted using R software (version 4.3.1; R Foundation for Statistical Computing, Vienna, Austria).

### 2.7. Statistical Analysis

A completely randomized design (CRD) was used for the whole study. All the experiments were conducted in triplicate, and the data were reported as mean ± standard deviation (SD). One-way analysis of variance (ANOVA) was done, and Duncan’s multiple range test (DMRT) was employed to compare the means. Statistical analysis was done using SPSS software package (SPSS 27.0 for Windows, SPSS Inc., Chicago, IL, USA).

## 3. Results and Discussion

### 3.1. Yield and Characteristics of CDs Derived from Red, Green and Brown Seaweeds

#### 3.1.1. Yield

Gravimetric yields of CDs derived from different seaweeds varied, depending on species. Among the different SWCDs, GS-CDs showed the highest yield (66.5%), followed by HD-CDs (60.65%), UL-CDs (60.5%), CP-CDs (57.5%), SP-CDs (57.0%), and SA-CDs (51.0%). The lowest yield was found for SA-CDs (51%). The differences in yield among all the CDs were plausibly attributed to the different chemical compositions among the varying seaweeds used. The red and green seaweeds mostly contain polysaccharides such as carrageenan, ulvan, and agar [32,33], which could be readily converted to CDs by the hydrothermal process. In contrast, the brown seaweed, composed of complex polysaccharides including fucoidan and alginate [34], might not undergo hydrothermal degradation completely. This led to the lower yield of CDs. Different yields of CDs were documented when various biomass sources were used as the raw materials of CDs [13,33].

#### 3.1.2. Appearance and TEM Images

**Figure 1:**
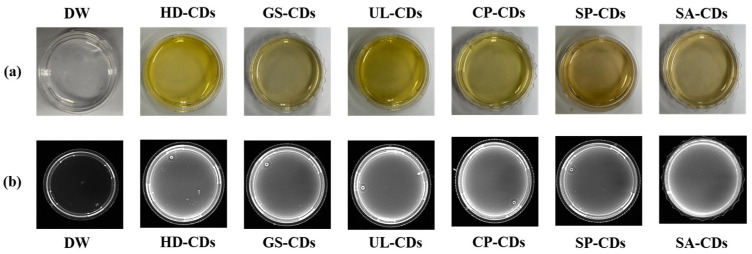
(**a**) Photographs under normal light and (**b**) UV light exposure of SWCDs. Abbreviations: DW: distilled water; SWCDs: seaweed-derived carbon dots; GS-CDs: *Gracilaria salicornia* carbon dots; HD-CDs: *Halymenia dilatata* carbon dots; UL-CDs: *Ulva lactuca* carbon dots; CP-CDs: *Caulerpa peltata* carbon dots; SP-CDs: *Sargassum polcystum* carbon dots; SA-CDs: *Spatoglossum asperum* carbon dots.

The CDs obtained were yellow–brownish in color Figure 1a. However, the degree of brownish color varied, depending on the seaweed used. This might be determined by indigenous pigments or components that underwent decomposition during the hydrothermal process. When exposed to UV light, all the SWCDs exhibited fluorescence emission when compared with distilled water t (Figure 1b). The brighter appearance of the SWCDs compared with distilled water under UV light exposure indicated the presence of fluorescence, confirming the successful formation of carbon dots [27].

**Figure 2:**
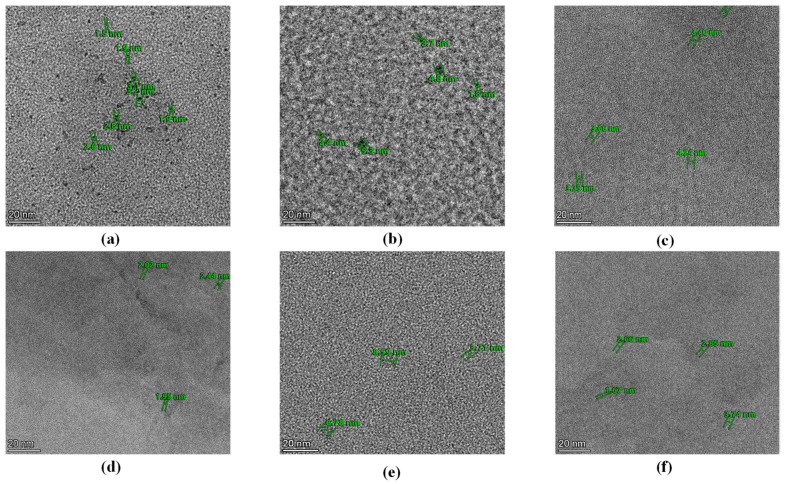
TEM images of SWCDs: (**a**) HD-CDs, (**b**) GS-CDs, (**c**) UL-CDs, (**d**) CP-CDs, (**e**) SP-CDs, (**f**) SA-CDs. Key: see Figure 1 caption.

The images captured by TEM revealed that all the CDs had a size less than 10 nm with spherical morphology (Figure 2). The present findings were in line with Li et al. [34], who reported the nano-size and spherical morphology of nitrogen- and sulfur-doped CDs from seaweed, synthesized using the hydrothermal process. In addition, similar results have been documented for CDs from different sources such as leaves [35], hair roots [36] and fruit peel [37] when the hydrothermal process was employed.

#### 3.1.3. SEM-EDX Spectra

**Figure 3:**
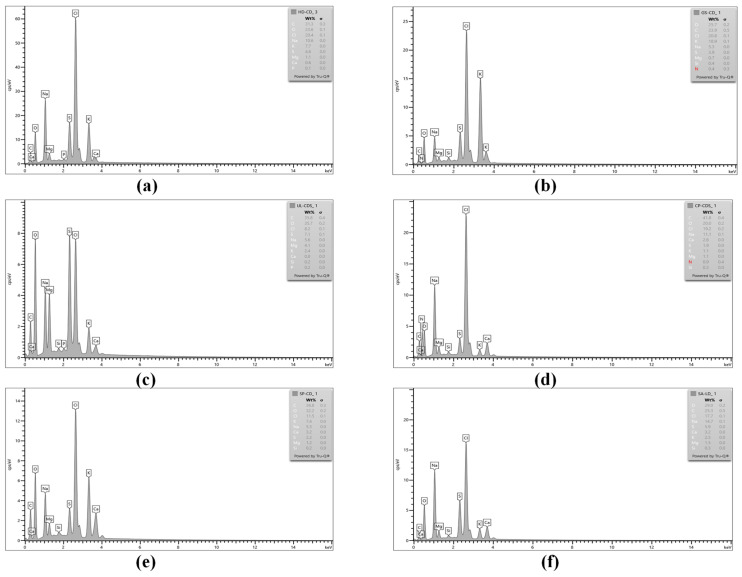
SEM EDX spectra of (**a**) HD-CDS, (**b**) GS-CDs, (**c**) UL-CDs, (**d**) CP-CDs, (**e**) SP-CDs, (**f**) SA-CDs. Key: see Figure 1 caption.

SEM-EDX spectral analysis of all the SWCDs showed different elemental compositions (Figure 3). Carbon and oxygen were found in all CDs samples, but their contents varied, depending on the seaweeds used. These components found in CDs were mainly attributed to the presence of hydrocolloids rich in carbon and oxygen in their molecules [38]. During the formation of CDs, these hydrocolloids, along with other biomolecules, contributed significantly to the formation of oxygen-containing surface functional groups. The presence of sulfur in the synthesized CDs might be due to the sulphated polysaccharide [39] present in the seaweeds. Sodium, potassium, calcium, phosphorus, and magnesium were present in seaweeds [40]. Those elements were also observed in the prepared CDs in the present study. It was noted that Cl was found in all CDs at high content. Seaweeds inhabiting the marine environment could absorb Cl in the form of NaCl from seawater. Those minerals might interact with functional groups or be localized individually on the surface of CDs, thus modifying the surface chemistry of the resulting CDs [41]. The presence of nitrogen was found only in CP-CDs and GS-CDs, which might be derived from the protein in seaweed during the hydrothermal process. Si was observed in all CDs, except HD-CDs. Si might be from the sand where the seaweed grew. The differences in the elemental compositions in varying CDs might be governed by different elemental profiles of various seaweeds, which were affected by species, environment, elemental contamination from the seawater, climate, etc. Overall, types of seaweed played a crucial role in determining the elements of the CDs, thereby influencing their surface properties.

#### 3.1.4. FTIR Spectra and XPS Spectra

**Figure 4:**
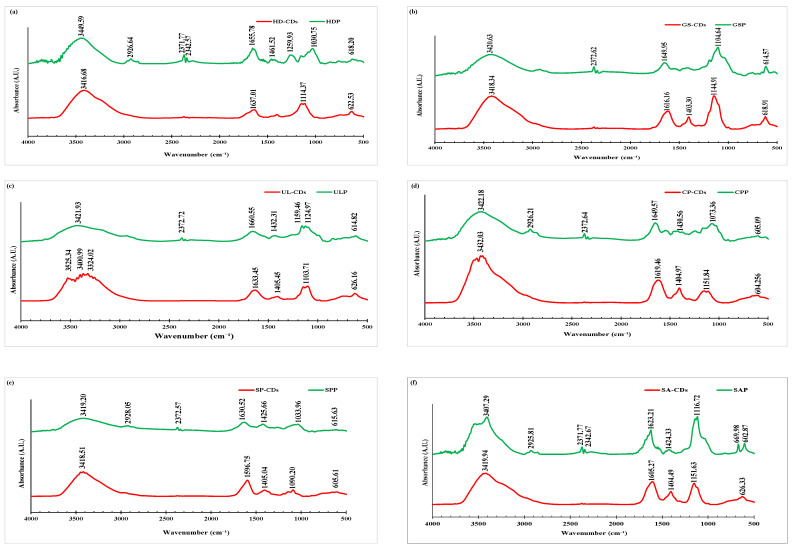
FTIR spectral images of (**a**) HD-CDs and *Halymenia dilatata* powder (HPP), (**b**) GS-CDs and *Gracilaria salicornia* powder (GSP), (**c**) UL-CDs and *Ulva lactuca* powder (ULP), (**d**) CP-CDs and *Caulerpa peltata* powder (CPP), (**e**) SP-CDs and *Sargassum polcystum* powder (SPP), (**f**) SA-CDs and *Spatoglossum asperum* powder (SAP). Key: see Figure 1 caption.

The FTIR spectra of raw seaweed powders and different SWCDs are presented in Figure 4. *Halymania dialtata* powder (HDP) demonstrated a broad band at 3449 cm^−1^, corresponding to the O-H stretching vibration. The result indicated the abundant hydroxyl group, mainly found in polysaccharides or hydrocolloids. The peak observed at 2926 cm^−1^ confirmed the presence of aliphatic C-H stretching of the methylene group. The vibration at 1461 cm^−1^ and 1259 cm^−1^ indicates the presence of sulfate ester groups and the S-O stretching vibration of sulphated polysaccharide [42]. Sulphated polysaccharides are found in all red, green and brown algae. Red algae consist of sulfated galactans; brown algae comprise fucoidan, while green algae contain ulvan-type sulphated polysaccharides [43]. The *Caulerpa peltata* powder (CPP) also exhibited similar vibrations corresponding to O-H, C-H stretching of methylene, C=C stretching of alkenes (1649 cm^−1^), and C-H bend of alkanes. The CP-CDs also exhibited similar peaks, suggesting that some functional groups were still retained after carbonization [44]. The ULP exhibited a broad O-H stretching peak at 3421.93 cm^−1^. The appearance of a prominent peak at 1660 cm^−1^ is attributed to the C=O stretching, and the band at 1432 cm^−1^ corresponds to aromatic C–C stretching. Additionally, the peak at 614 cm^−1^ is likely associated with the alkyl halide group [45]. For *Sargassum polysystum* powder (SPP) and *Spatoglossum asperum* (SAP), the peaks representing O-H, C=O, and C-O stretching were visible, and the results also aligned with the previous studies [46,47]. Although slight shifts and intensity changes were observed after hydrothermal reaction, most of the major peaks were retained in SWCDs, indicating the partial preservation of precursor chemical features.

**Figure 5:**
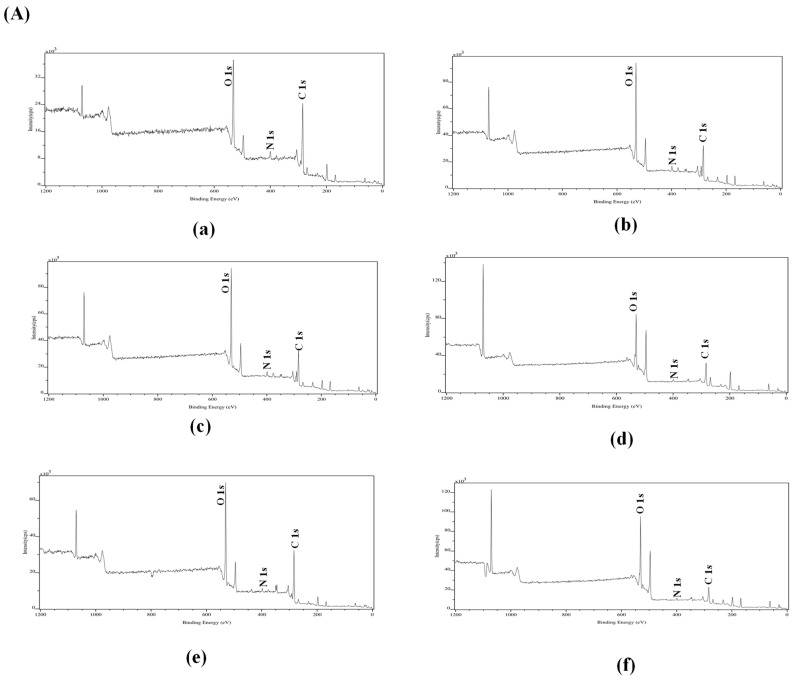

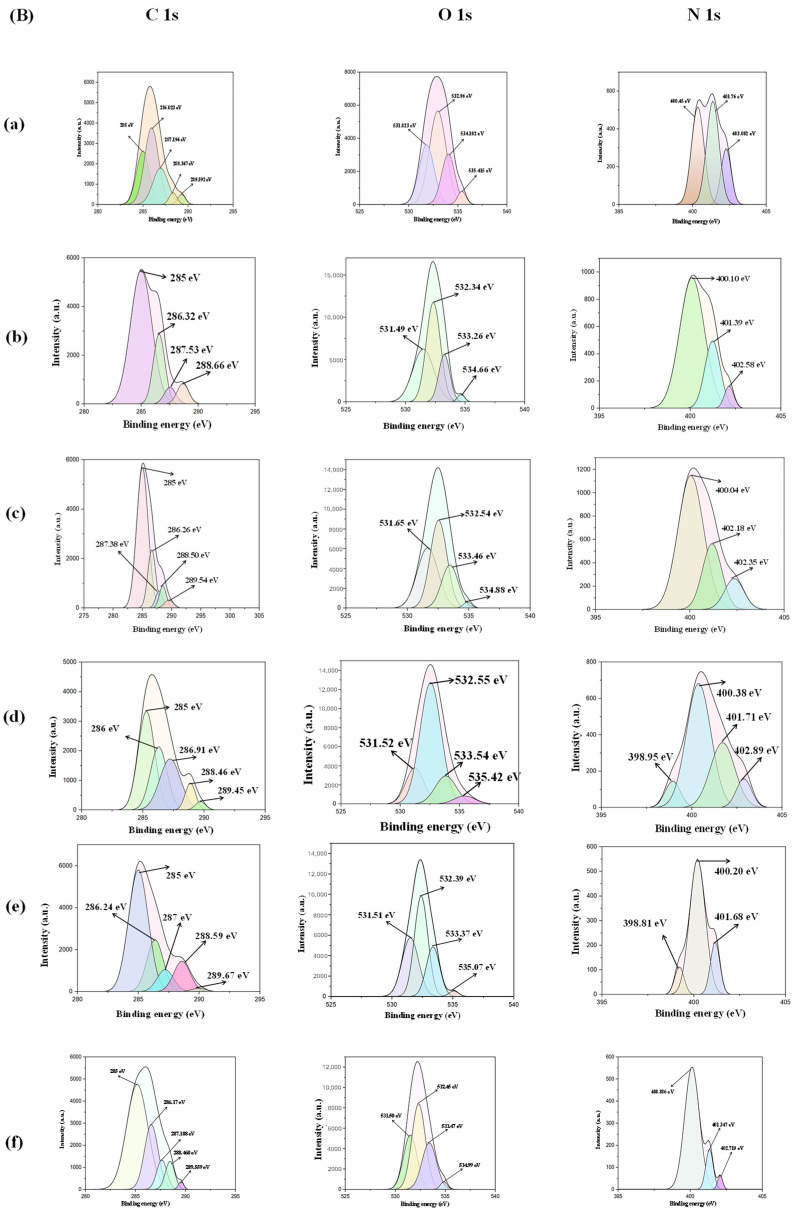
(**A**) Wide-scan XPS spectra and (**B**) High-resolution XPS spectra of C1s, O1s and N 1s of SWCDs: (**a**) HD-CDs, (**b**) GS-CDs, (**c**) UL-CDs, (**d**) CP-CDs, (**e**) SP-CDs, (**f**) SA-CDs. Key: see Figure 1 caption.

The surface elemental composition of SWCDs was also analyzed using XPS as shown in Figure 5A. The XPS spectra of SWCDs demonstrated the characteristic peaks at binding energies of approximately 1071 eV (Na 1s), 532 eV (O 1s), 400 eV (N 1s), 293 eV (K 2p), 285 eV (C 1s), 199 eV (Cl 2p) and 169 eV (S 2p), 50 eV (Mg 2p) 103 eV (Si 2p) 352 eV (Ca 2p) [48,49,50]. Different XPS spectra among HD-CDs, GS-CDs, UL-CDs, CP-CDs, SA-CDs, and SP-CDs thus indicated the presence of different elements such as Na, K, Ca, Cl, C, O, N, S, Si, and Mg. The elemental composition of SWCDs was expressed in atomic percentage (Table 1). HD-CDs and SP-CDs exhibited the highest carbon content, indicating comparatively greater carbonization, whereas SA-CDs showed the lowest carbon content and highest oxygen content, suggesting a greater abundance of oxygen-containing surface functional groups. The elevated levels of sodium observed in CP-CDs and SA-CDs might be associated with residual marine minerals retained during synthesis. The presence of sulfur and nitrogen was detected in all samples, indicating the incorporation of naturally occurring heteroatoms from seaweed biomass into the carbon framework. Kim et al. [49] also reported the presence of different elements in CDs from marine algae. The differences observed between XPS and EDX elemental compositions might be attributed to the different analytical principles and information depths between the two analytical techniques. XPS provides surface-sensitive elemental information from the outermost nanometers of the material, whereas EDX collects signals from a substantially larger interaction volume extending into the bulk; therefore, variations in elemental percentages are expected [51].

The dominant C 1s and O 1s peaks of SWCDs confirmed the formation of carbonaceous nanostructures enriched with oxygen-containing functional groups generated during carbonization of seaweed biomass. The high-resolution C 1s XPS spectrum of SWCDs was deconvoluted into five distinct peaks with varying intensities at binding energies of 285.0, 286.02, 287.2, 288.3, and 289.4 eV, which were assigned to C–C/C=C, C–O/C–N, C=O, O–C=O, and π–π* transitions, respectively (Figure 5B). The predominant intensity of the peak centered at 286.023 eV indicated the abundance of oxygen-containing surface as well as hydroxyl and ether groups on the surface of the CDs [52]. Furthermore, the high-resolution O 1s XPS spectrum exhibited multiple deconvoluted peaks at binding energies of 531.823, 532.96, 534.102, and 535.415 eV, corresponding to C=O, C–O, C–O–C, and -COOH, respectively [28]. In addition to the C 1s and O 1s peaks, the N 1s peak of the SWCDs was deconvoluted into pyridinic N (398.81–398.95 eV), pyrrolic N (400.04–400.61 eV), graphitic N (401.04–401.71 eV), and oxidized N (402.19–402.89 eV) [53,54]. The prevalence of pyrrolic-N across all CD variants suggested that the native protein and amino acid content of the seaweed biomass served as an effective nitrogen source, successfully cyclizing into the carbon core during hydrothermal treatment [55]. Interestingly, pyridinic-N was only detected in CP-CDs (398.95 eV) and SP-CDs (398.81 eV), suggesting that these two variants incorporated a higher degree of edge-plane nitrogen. In contrast, graphitic N was detected in HD-CDs, GS-CDs, UL-CDs, CP-CDs, and SP-CDs, confirming the successful incorporation of nitrogen into the carbon lattice during hydrothermal carbonization.

#### 3.1.5. UV–Vis Spectrophotometric and Spectrofluorometric Spectra

**Figure 6:**
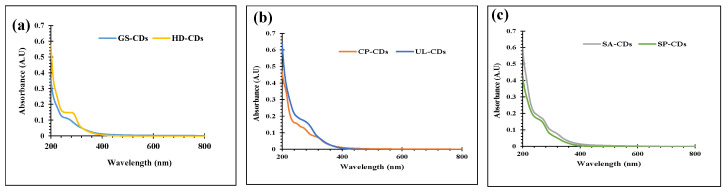
UV–visible absorption spectra of (**a**) HD-CDs and GS-CDs, (**b**) UL-CDs and CP-CDs, (**c**) SP-CDs and SA-CDs. Key: see Figure 1 caption.

Based on UV visible spectra of different SWCDs, all the CDs exhibited the maximum absorption in the range of 250–280 nm (Figure 6). Nonetheless, SA-CDs showed multiple peaks in the range of 260–330 nm. According to the previous studies conducted by Murugan et al. [27], the peaks observed at 200–300 nm represent the π–π* (sp^2^ aromatic/alkenyl C=C bond), and the peaks found at 320 nm were plausibly caused by the n−π* transition of C=O bonds. There were no peaks observed in the visible region, indicating the elimination of partial carbonization products [56].

**Figure 7:**
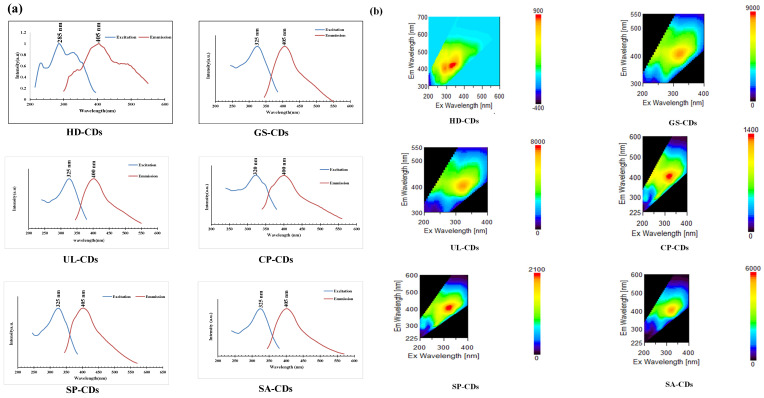
(**a**) 3D−fluorescence responses at different excitation wavelengths (200–400 nm) and emission wavelengths (225–550 nm), (**b**) fluorescence excitation and emission spectra of SWCDs. Key: see Figure 1 caption.

The photoluminescence spectra of all SWCDs were analyzed, and all of the SWCDs exhibited excitation-dependent fluorescence, a characteristic of CDs [57]. All of the samples except HD-CDs (285 nm) showed an excitation maximum at the wavelength of 320–325 nm (Figure 7). The maximum emission of all SWCDs was in the range of 400–405 nm. As the excitation wavelength increased, the emission peak was gradually shifted to a longer wavelength. The emission behavior of the CDs was affected by differences in the composition of the precursor [10]. The higher degree of graphitization and rich oxygen functionalization of HD-CDs, evidenced by the XPS and FTIR analysis, yielded the highest photoluminescence intensity alongside a blue-shifted excitation maximum (285 nm) [58]. In general, the excitation-dependent photoluminescence of CDs is predominantly governed by surface functional groups C–OH, C–O–C, C=O, and C–H, which introduce multiple emissive trap states within the sp^2^ conjugated (C=C) domains. Consequently, varying the excitation wavelengths activates distinct surface states, resulting in tunable emission characteristics [59]. The natural fluorescence properties of CDs make them promising materials for future applications, particularly in bioimaging and sensing.

#### 3.1.6. UV-Blocking Property

**Figure 8:**
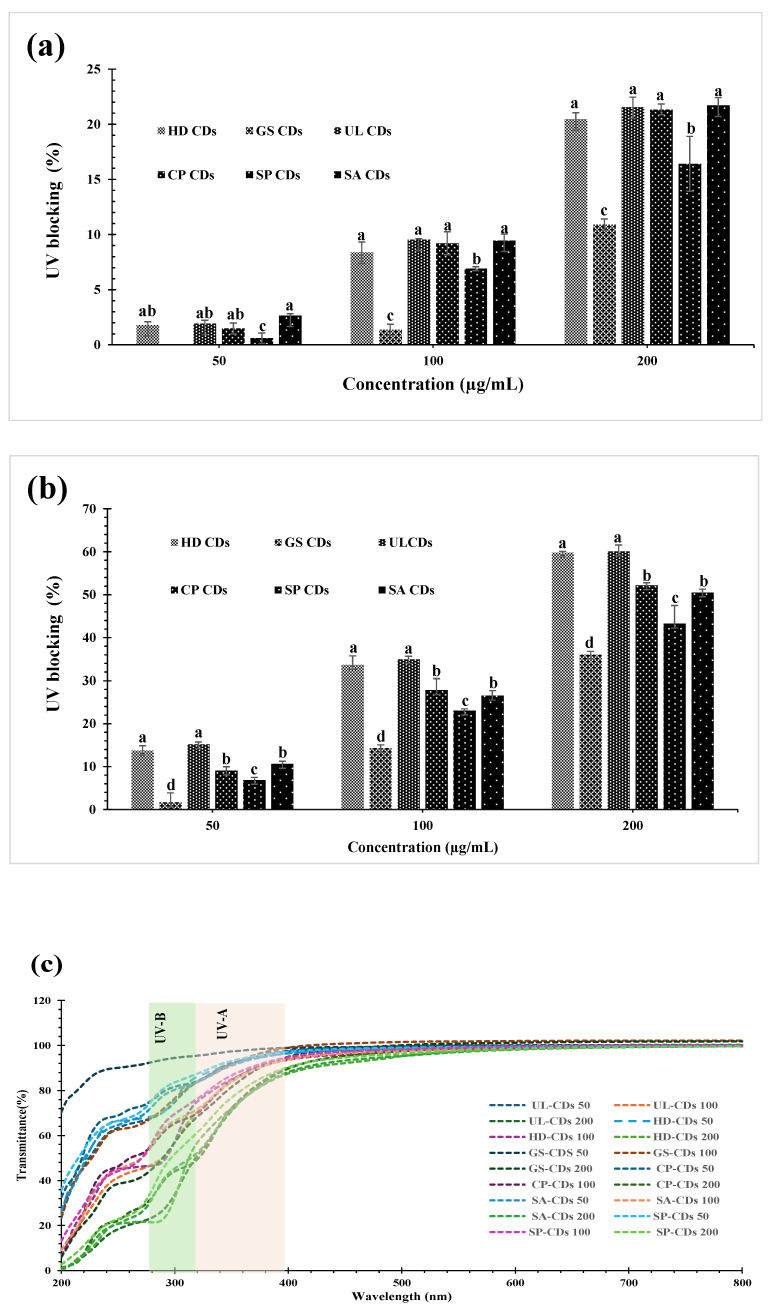
(**a**) % UV-A (320–400 nm), (**b**) % UV-B (280–320 nm) blocking properties and (**c**) UV–visible light transmittance spectra of SWCDs at various concentrations (50, 100 and 200 µg/mL). Different colored lines represent the UV–visible transmittance spectra of the different SWCDs at concentrations of 50, 100, and 200 µg/mL. Vertical bars represent the standard deviation (*n* = 3). For each UV light source, different lowercase letters on the bars within the same concentration of different CDs indicate significant differences (*p* < 0.05). Key: see Figure 1 caption.

The SWCDs exhibited notable light-barrier properties, with the lowest radiation transmittance observed in the range of 280–320 nm rather than 320–480 nm. UV radiation can induce the oxidation of proteins and lipids, thereby enhancing chemical deterioration, especially undesirable off-odors [24]. UV-blocking efficacy highlighted the potential of these CDs for future applications in active packaging designed to protect fatty foods during light-exposed retail displays. The strongest absorption was observed due to the UV-A and UV-B blocking abilities, which increased with rising concentrations of all CDs tested. HD-CDs and UL-CDs showed higher UV-B blocking capability than other CDs (*p* < 0.05), regardless of concentrations (Figure 8). GS-CDs showed very low blocking activity at all concentrations, especially at low concentration (50 µg/mL) (*p* < 0.05). The higher blocking efficiency of HD-CDs and UL-CDs was due to the higher atomic concentration of nitrogen (3.75% and 3.29%, respectively) than the less effective SP-CDs (1.93%). The heteroatom doping, along with the abundant oxygen-rich functional groups such as carboxyl and carbonyl groups and high degree of carbonization in the HD-CDs, favored the enhanced π–π* transmission of C=C and n–π* transition of C=O bonds [60]. GS-CDs, despite containing nitrogen, likely possess a less favorable combination of conjugated sp^2^ domains and surface defect states, resulting in weaker UV absorption and consequently lower UV-blocking efficiency.

For UV-A blocking capability, higher concentration demonstrated higher shielding of UV-A radiation, compared with lower concentration. At the same concentration, HD-CDs, UL-CDs, CP-CDs and SA-CDs showed higher blocking ability than GS-CDs and SP-CDs (*p* < 0.05). Nonetheless, there was no difference among all four formers CDs (*p* > 0.05). A similar result was observed to that of UV-B blocking ability, in which GS-CDs had the lowest capacity in blocking UV-A (*p* < 0.05). This observation could be explained by Beer–Lambert’s law, stating that absorbance was directly proportional to the concentration of absorbing species [61]. Overall, higher UV absorption was related to the reduction in light transmittance, thereby enhancing the UV-blocking capability. Among all the samples, CDs prepared from both species of green seaweeds, UL-CDs and CP-CDs, comparatively showed stronger UV-blocking ability. Interestingly, HD-CDs and SA-CDs from red and brown seaweed, respectively, also had stronger activity, but the other species in both groups showed much lower blocking activity. The observed interspecies variation indicated that the chemical composition of each seaweed strongly influenced the functional properties of CDs, including UV blocking ability. The differences in composition in seaweeds were mainly affected by the environmental conditions, geographical location, and growth habitat [62]. Furthermore, the differences in thermal stability and transformation of seaweed polysaccharides, pigments, and polyphenols during the hydrothermal process might contribute to the variations in the functional groups possessing UV-blocking capability of synthesized CDs [63].

### 3.2. Antioxidant and Antimicrobial Activities of Different SWCDs

#### 3.2.1. Antioxidant Activities

The antioxidant competency of different SWCDs as assayed by DPPH-RS-A, ABTS-RS-A, FRA-P, and MC-A is shown in Table 2. All the synthesized CDs showed free radical scavenging and reducing activities in a dose-dependent manner (125–500 µg/mL).

SWCDs with the ability of proton donation could stabilize the DPPH radical as shown by DPPH-RS-A (Table 2). At low concentration (125 µg/mL), HD-CDs had the highest DPPH-RS-A (*p* < 0.05). When the concentration of 250 µg/mL was used, HD-CDs and SP-CDs showed higher activity than others (*p* < 0.05). At 500 µg/mL, HD-CDs, UL-CDs, SP-CDs, and SA-CDs showed similar DPPH-RS-A (*p* > 0.05) but had higher activity than the rest (*p* < 0.05). At higher concentration (500 μg/mL), the availability of redox-active surface sites increased, allowing more hydrogen atom and electron transfer. The FTIR and XPS analysis indicate that the SWCDs are enriched with oxygen-containing functional groups, including hydroxyl(-OH), carboxyl (-COOH), and carbonyl (C=O), together with aromatic sp^2^ carbon domains. These functional groups serve as hydrogen- and electron-donating sites while conjugated sp^2^ domains stabilize the resulting radicals through electron delocalization [64]. Furthermore, the naturally incorporated nitrogen functionalities, particularly pyrrolic and graphitic nitrogen, can increase the electron density and facilitate the electron transfer reaction. The combined effect of these structural features enhances the radical scavenging ability of CDs [65]. CDs synthesized from different biomass sources like *Yongchuan xiuya* tea and coriander leaves also demonstrated DPPH-RS-A [66,67].

At all the tested levels, SA-CDs exhibited the highest ABTS-RS-A, compared to others (*p* < 0.05). Conversely, UL-CDs exhibited the lowest ABTS-RS-A at 250 and 500 µg/mL (*p* < 0.05). The ABTS-RS-A primarily estimates the antioxidant activity through an electron transfer mechanism, in which the ABTS radicals were transferred into ABTS^+^ radicals [68]. The relatively high sulfur content along with the higher oxygenated surface of SA-CDs might have further enhanced ABTS radical scavenging by modifying the surface electronic properties of CDs and facilitating electron transfer during the reduction in ABTS^+^ radicals [69]. Basically, ABTS-RS-A has been used to test the antioxidant activity of amphiphilic antioxidants, which are mainly localized in the aqueous phase [69]. Meng et al and Gedda et al. [70,71] also reported that CDs from banana peels and leaves of *Azadirachta indica* showed ABTS-RS-A, in which the activity depended on the concentrations used. As a result, SA-CDs were effective in preventing oxidation in less hydrophobic food systems.

On the other hand, the FRA-P assay evaluates the antioxidant potential by reducing Fe^3+^ to Fe^2+^ without the involvement of free radicals [72]. HD-CDs had the highest FRA-P at all concentrations tested (*p* < 0.05), compared to other SWCDs. CP-CDs had the lowest FRA-P at the highest concentration (500 µg/mL) (*p* < 0.05). The higher FRA-P value observed for HD-CDs might be attributed to the presence of higher carbon content, indicating a higher degree of carbonization and formation of well-developed sp^2^ conjugated carbon domains that facilitate electron transfer. In addition, the abundance of oxygen-containing functional groups and graphitic nitrogen identified by FTIR and XPS provide electron-donating sites and enhance the reduction of Fe^3+^ to Fe^2+^, resulting in greater ferric reducing antioxidant power [73]. The potential to donate electrons was confirmed by ABTS-RS-A. Tanna et al. [74] reported that the ethanolic extract of SA rich in flavonoid and phenolic compounds demonstrated high antioxidant activity. Zhang et al. [66] found that CDs from *Yongchuan xiuya* tea had high FRA-P, indicating the ability to provide electrons to free radicals, in which the propagation stage could be impeded.

The strongest metal chelation activity was observed for HD-CDs at 500 µg/mL (*p* < 0.05). However, similar MC-A was found between HD-CDs and SP-CDs at low concentration (125 µg/mL). At higher concentration, the synergistic effects of abundant surface functional groups, including hydroxyl, carboxyl, and carbonyl groups, together with a highly graphitic structure and small particle size, likely contribute to the significantly higher metal-chelating activity of HD-CDs compared with the other SWCDs. CDs from German chamomile flower [30] and cashew and jik leaves [13] were reported to possess MCA, which could help decrease lipid oxidation at the initial stage. Metal ions have been known to act as prooxidants, accelerating lipid oxidation [75]. Olasehinde et al. [76] also confirmed the potential of seaweed extract in chelating the Fe^2+^ ions, highlighting their ability to bind with transition metals, thereby lowering metal-catalyzed oxidative reactions. SWCDs had varying antioxidant activities with different modes of action, depending on the composition of seaweed used for the hydrothermal process. Thus, varying activities were mainly governed by the types of seaweeds used as precursors for CD synthesis.

#### 3.2.2. Antifungal Activity

**Figure 9:**
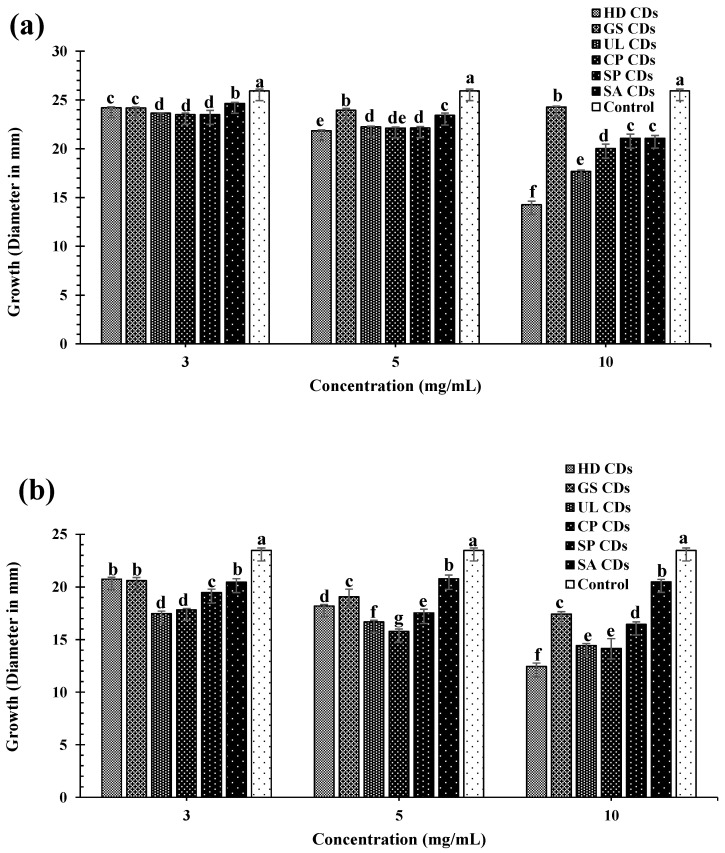
Growth of *Aspergillus parasiticus* (**a**) and *Aspergillus flavus* (**b**) as affected by treatment with different seaweed-derived carbon dots at various concentrations (3–10 mg/mL). Their growth was reported in terms of diameter (mm). Vertical bars represent the standard deviation (*n* = 3). Different lowercase letters on the bar within the same concentration of different seaweed-derived CDs indicate significant differences (*p* < 0.05). Key: see Figure 1 caption.

The antifungal activity of varying SWCDs was evaluated against *Aspergillus flavus* (AF) and *Aspergillus parasiticus* (AP), which are widely associated with the contamination and spoilage of various foods, particularly dried or semi-dried foods, during processing and storage [77]. All the SWCDs showed an inhibitory effect on the growth of AF and AP on day 3 of incubation (Figure 9). The HD-CDs showed the greatest reduction in the growth of AP when treated at 5 and 10 mg/mL (*p* < 0.05). At 10 mg/mL, the highest inhibition was found (*p* < 0.05). At low concentration (3 mg/mL), no obvious differences in zone inhibition were found for most SWCDs, while the control showed the highest growth as indicated by the largest diameter. For AF, HD-CDs also showed the highest inhibition, compared to other SWCDs (*p* < 0.05), followed by UL-CDs and CP-CDs. On the other hand, at lower concentrations (3 and 5 mg/mL), CP-CDs had the highest inhibition toward AF (*p* < 0.05). The results suggested that CDs from different seaweeds exhibited varying fungal inhibition efficacy, depending on the concentration used.

In addition, the differences in antifungal activity among all CDs from different seaweeds might be governed by different factors, including the size, functional groups on the CD surface, and composition of CDs [78]. The antifungal activity observed in the present study might be attributed to the unique physicochemical characteristics of individual CDs, including their ultra-small size, large surface area-to-volume ratio, and abundance of surface functional groups [78]. The ultra-small size of CDs allows them to penetrate cell walls of fungi and induces oxidative stress, ultimately leading to the death of the organism [79]. Antifungal mechanisms involving membrane disruption, ROS overproduction, mitochondrial depolarization, and intracellular metabolic collapse have been reported for functionalized CDs against AF [80]. CDs from different sources, including lemon and onion juices, were shown to have antifungal activity toward different strains [81]. Genovese et al. [82] documented that the red seaweed extract exhibited antifungal activity against *Aspergillus* spp., associated with the presence of bioactive compounds such as bromophenol and sulphated polysaccharides. Farghl et al. [83] reported antifungal activity of ethanolic extracts of red, brown, and green algae and observed potential inhibitory activity of those extracts. The synergistic effects of abundant nitrogen, sulfur, and oxygen-containing surface functional groups, favorable graphitic (sp^2^) carbon domains, and small particle size were more likely to determine the antifungal activity of SWCDs [84].

#### 3.2.3. Antibacterial Activity

SWCDs showed antibacterial activity against three pathogenic bacteria (*Listeria monocytogenes*, *Staphylococcus aureus,* and *Escherichia coli*), commonly found in seafood, and two spoilage bacteria (*Shewanella putrefaciens* and *Pseudomonas aeruginosa*), which are the major causes of seafood spoilage [85]. All the SWCDs demonstrated inhibition of bacterial growth and displayed MIC values ranging from 15.625 to 125 mg/mL. Among all SWCDs, SP-CDs and SA-CDs exhibited stronger antibacterial activity against all the pathogenic strains with MIC values ranging from 15.62 to 62.5 mg/mL (Table 3). The structural synergy plausibly enhanced the affinity of SP-CDs and SA-CDs toward the bacterial envelope.

The abundant amine and oxygen-based functional groups evidenced by the characterization studies initiated electrostatic adsorption to the bacterial surface, which was particularly effective against the negatively charged outer membranes [86]. Consequently, the graphitic domains were allowed to efficiently destabilize membrane integrity. There was no significant difference in the MIC values of SWCDs against spoilage bacteria, and all the CDs showed the same MIC values (62.5 mg/mL), except UL-CDs and SA-CDs, which had higher MIC values toward *Pseudomonas aeruginosa*. CDs from banana peel waste and *Impatiens balsamina* stems were demonstrated to inhibit both spoilage and foodborne pathogens [70,87]. Kumaravelu et al. [88] demonstrated that the strong antibacterial effect was observed for the ethyl acetate extracts of *Ulva lactuva* against different bacterial strains.

The antibacterial activity of all CDs was higher against Gram-positive bacteria rather than Gram-negative strains. Gram-negative microorganisms have a protective outer membrane with lipopolysaccharide, phospholipid, and lipoproteins, which are impermeable to foreign molecules [89,90]. The antibacterial activity of different seaweed extracts was also confirmed by earlier research conducted by Hejna et al. [91], which suggested that the antibacterial properties were due to the presence of functional groups that interact with the bacterial cell wall at different levels and the suppression of oxidative phosphorylation. These processes increase the cytoplasmic membrane’s permeability, which destroys cell membranes, inhibits enzymes, intercalates DNA, and causes cell lysis [92]. The antibacterial activity of CDs from seaweed could be improved by doping with polyphenols such as epigallocatechin gallate, quercetin, and caffeic acid, which can enrich the oxygen-containing functional groups, and also by doping with heteroatoms, e.g., boron and nitrogen [93,94,95].

### 3.3. Cytotoxicity

**Figure 10:**
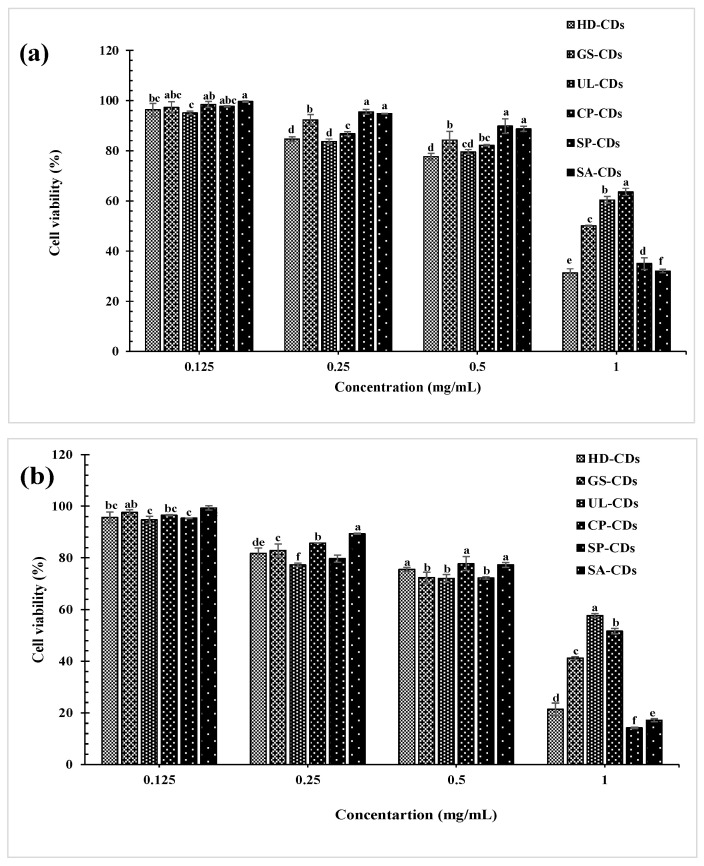
Cell viability of BJ cells after treatment with SWCDs at different concentrations (0.125–1 mg/mL) for 24 (**a**) and 48 h (**b**). Vertical bars represent the standard deviation (*n* = 3). Different lowercase letters on the bars within the same concentration of different seaweed-derived CDs indicate significant differences (*p* < 0.05). Key: see Figure 1 caption.

The biocompatibility of CDs is an important factor determining the applicability of CDs in different sectors including pharmaceuticals [96] and food [97]. The cytotoxicity of all SWCDs was tested on BJ cells at concentrations of 1, 0.5, 0.25, and 0.125 mg/mL using the MTT assay over 24 and 48 h of exposure, as presented in Figure 10. All the CDs exhibited cytotoxic effect at a concentration of 1 mg/mL, and the lower concentrations of 0.50, 0.25, and 0.125 mg/mL showed a cell viability greater than 70%, which is considered the threshold limit for biocompatibility [98]. All the SWCDs displayed above 90% cell viability at a lower concentration (0.125 mg/mL). At a higher concentration (1 mg/mL), red and brown seaweeds showed more cytotoxicity towards BJ cells after 48 h treatment. Although CDs synthesized from green seaweeds exerted cytotoxicity at 1 mg/mL, they maintained a higher percentage of cell viability (60% and 63%) when exposed for 24 h in comparison with CDs derived from red and brown seaweeds. Following 48 h of exposure, CDs at concentrations of 0.125, 0.25 and 0.5 mg/mL did not show major changes in cell viability. In contrast, treatment with a higher concentration of 1 mg/mL on BJ cells exerted a potential cytotoxic effect with longer exposure time, suggesting a dose-dependent cytotoxicity of the SWCDs on BJ cells.

The changes in cell viability might be attributed to the difference in the surface chemistry of CDs as evidenced by XPS, FTIR, and EDX spectral analyses. The variations in chemical composition of red, green and brown seaweed could influence the formation of distinct surface functional groups. These differences consequently led to the observed changes in cell viability [30]. The reduced cell viability observed following prolonged exposure to higher concentrations of SWCDs might be attributed to the increased interactions between the CDs and cellular components, leading to cellular stress and impaired metabolic activity [99]. The MTT results indicated that SWCDs at concentrations up to 0.5 mg/mL showed no significant cytotoxic effect towards BJ cells.

Despite exhibiting toxicity at higher concentrations, the MIC values of CDs remained above the concentration limit employed for preservation. The maximum limit of food preservatives in seafood restrict their antimicrobial applicability. Nonetheless, the SWCDs gained excellent antioxidant activity even at lower concentrations. Thus, considering the growing interest in replacing synthetic antioxidants with sustainable alternatives, CDs were applied to retard lipid peroxidation during autoxidative deterioration of shrimp lipids.

SWCDs exhibited toxicity at higher concentrations (1 mg/mL), and their minimum inhibitory concentrations (MICs; 15–125 mg/mL) exceeded the toxic threshold. Nonetheless, significant antioxidant activity was found at lower concentrations (125–500 µg/mL). Therefore, SWCDs were focused on being used as antioxidants to retard lipid oxidation and enhance oxidative stability in fatty foods [100]. Accordingly, their efficacy in retarding lipid auto-oxidation in shrimp was further evaluated, while chloramphenicol was incorporated solely to suppress microbial growth and eliminate microbial interference during storage to avoid the microbial action on lipid oxidation.

### 3.4. Effect of the Selected SWCDs on Prevention of Lipid Oxidation of Peeled and Deveined Pacific White Shrimp During the Refrigerated Storage

#### 3.4.1. PV and TBARS

**Figure 11:**
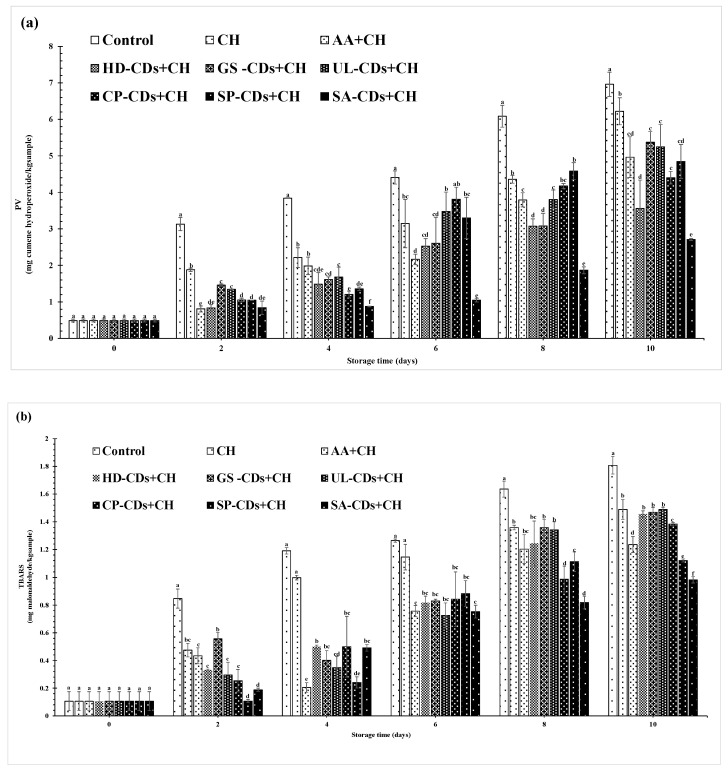
(**a**) The peroxide value and (**b**) TBARS value of ready-to-cook shrimp treated with SWCDs + chloramphenicol (CH) at a concentration of 500 ppm for 10 days. Vertical bars represent the standard deviation (*n* = 3). Different lowercase letters on the bar within the same storage time of different seaweed-derived CDs indicate significant differences (*p* < 0.05). Key: see Figure 1 caption.

The impact of different SWCDs on the oxidation of peeled and deveined Pacific white shrimp (PD-PWS) during the refrigerated storage of 10 days is shown in Figure 11. For all samples, except the control, microbial action was terminated by applying chloramphenicol, considered a strong antibiotic used against both Gram-positive and Gram-negative bacteria [101]. As a result, the effect of SWCDs against lipid oxidation was established without the interfering effect caused by microorganisms. PV and TBARS of PD-PWS meat during the refrigerated storage period of 10 days are presented in Figure 11. On day 0, all samples exhibited an initial PV of 0.49 and TBARS value of 0.10, indicating the freshness of the samples at the beginning of storage [102]. During refrigerated storage, the control samples without any treatment showed a gradual increase in PV and TBARS values, indicating the formation of hydroperoxides. Hydroperoxides are highly unstable compounds that undergo decomposition into secondary oxidation products such as aldehydes and ketones, which are responsible for the development of undesirable odor in seafood products [103].

The polyunsaturated fatty acids present in the shrimp are highly susceptible to oxidative deterioration [104]. PD-PWS treated with the SWCDs had the lowest PV when compared to the control samples during storage. Lipid peroxidation is a free radical-mediated chain reaction involving initiation, propagation, and termination stages. Reactive oxygen species such as hydroxyl radicals abstract hydrogen atoms from unsaturated fatty acids, leading to the formation of lipid peroxyl radicals, which further propagate the oxidation process by reacting with neighboring lipid molecules [105]. The antioxidant potential of SWCDs helped prevent oxidative degradation by scavenging reactive oxygen species [12]. In addition to direct radical scavenging, SWCDs might retard lipid oxidation through chelating Cu released due to the degradation of hemocyanin of PD-PWS. Fenton-mediated hydroxyl radical generation could be prevented to some extent, thus retarding lipid peroxidation [106]. The inhibition of oxidation of Pacific white shrimp was found when treated with soursop leaf extract [32]. The PD-PWS treated with chloramphenicol also exhibited a slightly lower PV value than the control. This confirmed that microorganisms were plausibly involved in the induction of lipid oxidation by their lipase, phospholipase, or lipoxygenase activities, etc. Those enzymes might be associated with the enhanced lipid oxidation [107].

Among all these treatments, SA-CDs showed an excellent radical scavenging activity, thereby reducing the lipid peroxidation more effectively. Similar trends were also observed in TBARS, where all SWCDs-treated samples showed lower formation of secondary lipid oxidation products than the control throughout the storage period. The result confirmed the effective inhibition of lipid peroxidation in PD-PWS by SWCDs. During the storage period, the samples treated with UL-CDs, HD-CDs, and GS-CDs exhibited similar TBARS values in the presence of chloramphenicol. However, SA-CDs- and SP-CDs-treated samples maintained significantly lower TBARS even on day 0, demonstrating excellent and sustained protection against oxidative deterioration during prolonged refrigerated storage. The SWCDs therefore retarded lipid peroxidation in PD-PWS, plausibly by terminating the oxidative chain reaction through ROS scavenging and metal chelation.

#### 3.4.2. Fatty Acid Profile

The fatty acid profiles of fresh PD-PWS at day 0, control, DW + CH, AA + CH, and SA-CDs + CH at day 10 are shown in Table 4. Saturated fatty acids (SFA), monounsaturated fatty acids (MUFA), and polyunsaturated fatty acids (PUFA) were identified in all treatment groups. Palmitic acid, linoleic acid, eicosapentaenoic acid (EPA), and docosahexaenoic acid (DHA) were the major fatty acids. The findings indicated that considerable changes were observed among untreated control, DW + CH, AA + CH, and SA-CDs + CH-treated PD-PWS during the 10-day storage period. At day 0, the control samples possessed a high proportion of PUFA (55.37 g/100 g lipid), followed by SFA (37.66 g/100 g lipid) and MUFA (6.97 g/100 g lipid), indicating the naturally high unsaturated fatty acids of shrimp. The predominant SFA was palmitic acid (19.59 g/100 g lipid), while linoleic acid, EPA, and DHA represented the major unsaturated fatty acids.

After 10 days of storage, the untreated control exhibited substantial changes in lipid composition. SFA increased (53.11 g/100 g lipid), whereas the PUFA decreased markedly from 55.37 to 40.17 g/100 g lipid. MUFA remained relatively unchanged. Among the individual fatty acids, EPA decreased from 12.96 to 9.23 g/100 g lipid, and DHA declined from 12.55 to 9.53 g/100 g lipid. Linoleic acid also showed a reduction from 21.86 to 15.52 g/100 g lipid. These changes indicated the progressive deterioration of unsaturated fatty acids during storage, especially for the control group.

DW + CH showed a similar fatty acid profile when compared with the untreated control on day 10. Therefore, the microorganisms present in the PD-PWS did not play a major role in enhancing the lipid oxidation during storage. Instead, auto-oxidation was dominant and became the key reaction associated with the changes in fatty acids in the samples, as shown in Table 4. In addition, the fresh sample was used in the present study, and a lower load of microorganisms was postulated. In general, microbial lipases and phospholipases are responsible for hydrolysis and subsequent oxidation of unsaturated fatty acids during storage [108].

Both treatments reduced lipid deterioration compared with the untreated control. AA + CH preserved PUFA (51.32 g/100 g lipid) and maintained EPA (12.54 g/100 g lipid) and DHA (11.79 g/100 g lipid), indicating suppression of oxidative degradation. SA-CDs + CH showed the greatest protective effect, retaining PUFA to a greater extent (52.78 g/100 g lipid) and limiting SFA accumulation (40.83 g/100 g lipid). EPA (12.73 g/100 g lipid) and DHA (12.14 g/100 g lipid) remained close to the initial values. The improved preservation of lipids by SA-CDs might be attributed to the antioxidant activity of SA-CDs. The abundant surface functional groups and electron-transfer capacity of CDs enabled hydrogen donation and radical neutralization, thereby limiting peroxidation of fatty acids and preserving the overall fatty acid profile during storage [100]. CDs have been extensively used for the preservation of perishable foods due to their higher ability to scavenge free radicals. CDs have been incorporated into packaging systems to extend the shelf life of highly perishable foods [109,110]. Retention of EPA and DHA in the treatment group could reflect the reduced oxidative damage and maintenance of nutritional quality during storage.

### 3.5. PCA Biplot

**Figure 12:**
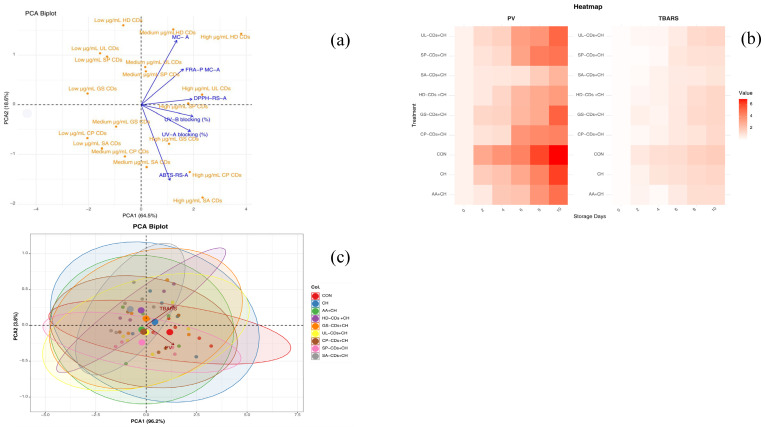
Correlation matrix heatmap and principal component analysis (PCA) illustrating the multivariate relationships among spoilage indices, TBARS, and PV of shrimp subjected to different treatments during refrigerated storage. (**a**) PCA biplot showing relationship between antioxidant and UV-blocking properties of CDs at different concentrations. (**b**) Correlation matrix heatmap illustrating correlations among quality parameters. (**c**) PCA biplot showing sample distribution and variable loadings. Abbreviations: CON: control; CH, chloramphenicol; AA, ascorbic acid; GS-CDs: *Gracilaria salicornia* carbon dots; HD-CDs: *Halymenia dilatata* carbon dots; UL-CDs: *Ulva lactuca* carbon dots; CP-CDs: *Caulerpa peltata* carbon dots; SP-CDs: *Sargassum polcystum* carbon dots; SA-CDs: *Spatoglossum asperum* carbon dots.

The PCA (PCA 1-64.5%, PCA 2-18.6%) was performed to evaluate the relationship between SWCDs at different concentrations, antioxidant activity, and UV-blocking efficacy (Figure 12a). The biplot clearly demonstrated the distinct clustering of SWCDs as influenced by their concentration and antioxidant activity. High concentrations of CDs were predominantly distributed along the positive PCA1 region, indicating the enhanced antioxidant and photoprotective properties at elevated concentrations.

Figure 12b illustrates the multivariate relationships among lipid oxidation indices and treatments of shrimp during refrigerated storage using a correlation matrix heatmap and principal component analysis (PCA). The correlation matrix heatmap (Figure 12b) demonstrated strong positive correlations among lipid oxidation-related parameters, indicating that lipid oxidation took place as indicated by the increases in PV and TBARS during storage. Parameters associated with lipid peroxidation exhibited clustering behavior, suggesting interconnected deterioration during refrigerated storage. The PCA biplot (Figure 12c) visualized the distribution pattern of samples and the contribution of quality indices to sample differentiation. PCA1 and PCA2 accounted for 96.2% and 3.8% of the total variance, respectively, explaining 100.0% of the cumulative variance. Samples gradually shifted from the negative toward the positive region of PCA1 with increasing storage time, reflecting progressive oxidative deterioration. The control sample exhibited the most rapid displacement toward positive PCA1 values, increasing from −1.74 at day 0 to 3.22 at day 10, indicating severe oxidative deterioration during storage. In contrast, all SWCD-treated samples showed slower PCA displacement, suggesting the delayed lipid oxidation and improved stability. Among them, SA-CDs + CH exhibited the strongest preventive effect toward lipid oxidation, with the lowest PCA1 value at day 10 (0.37), followed by HD-CDs + CH (1.37) and SP-CDs + CH (1.42). SA-CDs + CH and HD-CDs + CH samples remained clustered closer to the origin throughout storage, indicating suppression of lipid oxidation. The superior preventive performance of SA-CDs + CH might be attributed to enhanced radical scavenging activity and stronger interactions between SA-CDs functional groups and radicals, which could favor reducing power retard oxidative deterioration without the interfering impact from microorganisms. Although AA + CH effectively delayed oxidative deterioration during the early storage period, its PCA trajectory shifted more rapidly toward positive PCA1 values during prolonged storage compared with several SWCDs-treated samples. This finding suggested that SWCDs exhibited long-term protection against lipid peroxidation rather than AA for PD-PWS throughout the refrigerated storage.

## 4. Conclusions

Green synthesis of SWCDs likely retained key surface functional groups from their natural precursor to some extent. SWCDs exhibited notable bioactivities including antioxidant and antimicrobial activities, highlighting their potential as bioactive nanomaterials. Nonetheless, the physicochemical properties and bioactivities were determined by the seaweed used for CDs synthesis. The potential of SWCDs in preventing the lipid oxidation of perishable seafood products, particularly in peeled, deveined Pacific white shrimp under refrigerated storage conditions, provides a strong foundation for their application as a food antioxidant in seafood or fatty foods, in which the quality can be maintained during extended storage. Since SWCDs demonstrated antimicrobial activity, the application for shelf-life extension of seafoods or other perishable food products should be investigated. To enhance the efficacy of SWCDs for preservation, other technologies, especially non-thermal processing technologies, can be used in conjunction, in which optimal benefit can be achieved. However, this study presents a few limitations, as the SWCDs were evaluated within a single seafood matrix, in which their effectiveness may vary across other food systems. Furthermore, their sensory properties and long-term safety profiles were not assessed. Antimicrobial activity of SWCD should be improved by doping with safe compounds. These limitations highlight the critical need for further studies to confirm their broader applicability, in which sensory acceptance, and safety of treated products can be enhanced.

## Figures and Tables

**Table 1:** Surface atomic concentration of different elements of SWCDs obtained by XPS analysis.

Algae	Sample	Atomic Concentration (%)									
		Na	O	N	Ca	K	C	Cl	S	Si	Mg
Red	HD-CDs	3.78	25.63	3.75	ND	0.77	59.1	4.46	2.51	ND	ND
	GS-CDs	8.24	37.44	2.75	0.5	2.28	37.98	3.21	4.41	1.22	1.98
Green	UL-CDs	5.25	32.81	3.29	0.67	ND	48.35	1.29	4.07	1.49	2.77
	CP-CDs	20.5	28.99	2.24	0.7	ND	34.83	8.75	3.29	0.69	ND
Brown	SP-CDs	6.48	32.93	1.93	1.21	0.82	50.38	2.7	1.99	1.55	ND
	SA-CDs	19.67	40.29	1.83	0.74	ND	26.14	4.8	6.53	ND	ND

Abbreviations: GS-CDs, *Gracilaria salicornia* carbon dots; HD-CDs, *Halymenia dilatata* carbon dots; UL-CDs, *Ulva lactuca* carbon dots; CP-CDs, *Caulerpa peltata* carbon dots; SP-CDs, *Sargassum polycystum* carbon dots; SA-CDs, *Spatoglossum asperum* carbon dots; ND, not detected.

**Table 2:** Antioxidant activities of seaweed-derived carbon dots at different concentrations (125–500 µg/mL).

**Samples**	**DPPH-RS-A (µmol TE/L Sample)** **CDs Concentration (µg/mL)**			**ABTS-RS-A (µmol TE/L Sample)** **CDs Concentration (µg/mL)**		
	**500**	**250**	**125**	**500**	**250**	**125**
HD-CDs	148.32 ± 1.51 ^a^	142.78 ± 1.55 ^a^	117.84 ± 0.39 ^a^	561.40 ± 7.00 ^e^	366.40 ±10.00 ^e^	234.40 ± 2.00 ^f^
GS-CDs	126.03 ± 4.93 ^c^	89.14 ± 1.37 ^d^	71.93 ± 1.15 ^c^	867.40 ± 5.00 ^b^	746.40 ± 4.00 ^b^	523.40 ± 3.00 ^c^
UL-CDs	148.91 ± 2.98 ^a^	109.59 ± 4.43 ^c^	69.82 ± 1.51 ^c^	479.07 ± 5.03 ^f^	349.40 ± 5.00 ^f^	253.40 ± 5.00 ^e^
CP-CDs	135.70 ± 2.98 ^b^	86.59 ± 4.92 ^d^	65.47 ± 1.89 ^c^	802.40 ± 7.21 ^c^	650.40 ± 5.29 ^c^	541.07 ± 9.45 ^b^
SP-CDs	144.55 ± 1.71 ^a^	135.23 ± 8.41 ^a^	93.74 ± 8.96 ^b^	648.40 ± 2.00 ^d^	466.40 ± 5.29 ^d^	387.40± 1.00 ^d^
SA-CDs	144.66 ± 0.40 ^a^	126.98 ± 1.33 ^b^	88.57 ± 2.33 ^b^	1043.40 ± 3.00 ^a^	825.40 ± 7.00 ^a^	648.40 ± 8.00 ^a^
**Samples**	**FRA-P (µmol TE/L Sample)** **CDs Concentration (µg/mL)**			**MC-A (µmol EE/L Sample)** **CDs Concentration (µg/mL)**		
	**500**	**250**	**125**	**500**	**250**	**125**
HD-CDs	717.24 ± 7.87 ^a^	358.43 ±3.57 ^a^	131.29 ± 6.19 ^a^	56.12 ± 5.04 ^a^	50.72 ± 0.22 ^a^	48.38 ± 0.86 ^a^
GS-CDs	183.43 ± 11.43 ^c^	45.57 ± 3.57 ^c^	14.86 ± 1.43 ^e^	39.42 ± 0.48 ^c^	37.92 ± 0.33 ^d^	36.84 ± 0.23 ^c^
UL-CDs	181.29 ± 2.14 ^c^	88.67 ± 3.60 ^b^	38.19 ± 8.37 ^b^	47.61 ± 0.14 ^b^	46.33 ± 0.30 ^c^	42.46 ± 2.25 ^b^
CP-CDs	148.43 ± 0.71 ^d^	47.24 ± 3.60 ^c^	28.67 ± 2.97 ^d^	32.68 ± 1.78 ^d^	25.07 ± 0.33 ^f^	20.56 ± 1.53 ^d^
SP-CDs	205.33 ± 2.97 ^b^	91.52 ± 0.82 ^b^	48.19 ± 3.60 ^b^	47.61 ± 0.07 ^b^	47.43 ± 0.04 ^b^	46.20 ± 0.54 ^a^
SA-CDs	203.43 ± 1.43 ^b^	90.10 ± 3.60 ^b^	28.67 ± 3.30 ^d^	33.29 ± 2.47 ^d^	27.92 ± 1.09 ^e^	22.80 ± 2.52 ^d^

Note: Mean ± SD (*n* = 3). Different lowercase superscripts in the same column indicate significant differences (*p* < 0.05). Abbreviations: GS-CDs *Gracilaria salicornia carbondots;* HD-CDs, *Halymenia dilatata* carbon dots; UL-CDs, *Ulva lactuca* carbon dots; CP-CDs, *Caulerpa peltata* carbon dots; SP-CDs, *Sargassum polycystum* carbon dots; SA-CDs, *Spatoglossum asperum* carbon dots; ABTS-RS-A, ABTS radical scavenging activity; DPPH-RS-A, DPPH radical scavenging activity; FRA-P, ferric reducing antioxidant power; MC-A, metal chelating activity; TE, Trolox equivalent; EE, EDTA equivalent.

**Table 3:** Minimum inhibitory concentration (MIC) and minimum bactericidal concentration (MBC) of different seaweed-derived carbon dots against pathogenic and spoilage bacteria.

Samples	*Listeria monocytogenes*		*Staphylococcus aureus*		*Escherichia coli*		*Pseudomonas aeruginosa*		*Shewanella putrefaciens*	
	MIC (mg/mL)	MBC (mg/mL)	MIC (mg/mL)	MBC (mg/mL)	MIC (mg/mL)	MBC (mg/mL)	MIC (mg/mL)	MBC (mg/mL)	MIC (mg/mL)	MBC (mg/mL)
HD-CDs	62.5	62.5	62.5	62.5	62.5	125	62.5	125	62.5	125
GS-CDs	31.25	62.5	31.25	31.25	62.5	125	62.5	125	62.5	125
UL-CDs	62.5	125	62.5	62.5	125	125	125	125	62.5	125
CP-CDs	31.25	62.5	31.25	31.25	62.5	62.5	62.5	125	62.5	62.5
SP-CDs	15.62	31.25	15.62	15.62	62.5	62.5	62.5	62.5	62.5	62.5
SA-CDs	15.62	31.25	15.62	31.25	62.5	62.5	125	125	62.5	62.5

Note: All samples were analyzed in triplicate. Abbreviations: GS-CDs: *Gracilaria salicornia* carbon dots; HD-CDs: *Halymenia dilatata* carbon dots; UL-CDs: *Ulva lactuca* carbon dots; CP-CDs: *Caulerpa peltate* carbon dots; SP-CDs: *Sargassum polycystum* carbon dots; SA-CDs: *Spatoglossum aspermum* carbon dots.

**Table 4:** Fatty acid profiles of PD-PWS treated without treatment on day 0 and the selected treated samples stored at 4 °C for 10 days.

Fatty Acid (g/100 g Lipid)	Day 0	Day 10			
	Control	Control	DW + CH	AA + CH	SA-CDs + CH
C12:0 (Lauric)	ND	ND	ND	ND	0.45 ± 0.01
C14:0 (Myristic)	0.26 ± 0.03	0.29 ± 0.08	0.30 ± 0.05	ND	0.39 ± 0.02
C15:0 (Pentadecanoic)	1.28 ± 0.15	1.21 ± 0.40	1.54 ± 0.46	0.84 ± 0.58	0.91 ± 0.26
C16:0 (Palmitic)	19.59 ± 6.68	31.43 ± 8.48	29.02 ± 4.43	23.20 ± 0.68	23.70 ± 1.00
C17:0 (Heptadecanoic)	2.69 ± 1.92	3.90 ± 1.22	4.77 ± 1.39	4.69 ± 0.27	3.13 ± 0.83
C18:0 (Stearic)	13.54 ± 1.51	15.92 ± 4.30	12.32 ± 2.26	13.38 ± 0.50	11.96 ± 0.49
C20:0 (Arachidic)	0.30 ± 0.04	0.36 ± 0.10	0.21 ± 0.18	0.30 ± 0.01	0.29 ± 0.01
C14:1 (Myristoleic)	0.26 ± 0.03	0.34 ± 0.09	0.32 ± 0.05	0.24 ± 0.01	0.24 ± 0.01
C16:1 (Palmitoleic)	1.00 ± 0.11	1.34 ± 0.40	0.86 ± 0.21	0.77 ± 0.02	1.06 ± 0.18
C18:1 cis 9 (Oleic)	4.57 ± 0.51	4.00 ± 1.72	4.18 ± 0.81	4.29 ± 0.12	4.18 ± 0.19
C20:1 cis-11 (Eicosenoic)	1.15 ± 0.13	1.05 ± 0.48	1.08 ± 0.16	0.97 ± 0.10	0.91 ± 0.04
C18:2 cis 9,12 (Linoleic)	21.86 ± 2.44	15.52 ± 5.02	17.14 ± 2.67	19.07 ± 0.29	20.62 ± 0.74
C18:3 cis 6,9,12 (gamma-Linolenic)	1.33 ± 0.20	0.99 ± 0.50	1.28 ± 0.13	1.26 ± 0.23	1.07 ± 0.05
C20:2 cis 11,14 (Eicosadienoic)	2.85 ± 0.32	2.00 ± 0.65	2.86 ± 0.38	2.77 ± 0.09	2.74 ± 0.10
C20:3 cis 8,11,14 (Eicosatrienoic)	0.22 ± 0.11	ND	ND	ND	ND
C20:3 cis 11,14,17 (Eicosatrienoic)	ND	0.35 ± 0.10	ND	ND	ND
C20:4 cis 5,8,11,14 (Eicosatetraenoic)	3.60 ± 0.40	2.54 ± 0.84	2.98 ± 0.47	3.88 ± 0.11	3.48 ± 0.13
C20:5 cis 5,8,11,14,17 EPA (Eicosapentanoic)	12.96 ± 1.44	9.23 ± 2.97	10.74 ± 1.66	12.54 ± 0.19	12.73 ± 0.46
C22:6 cis 4,710,13,16,19 DHA (Docosahexaenoic)	12.55 ± 1.39	9.53 ± 2.91	10.41 ± 1.68	11.79 ± 0.18	12.14 ± 0.44
**SFA**	37.66 ± 10.31	53.11 ± 14.59	48.16 ± 8.88	42.41 ± 2.04	40.83 ± 2.63
**MUFA**	6.97 ± 0.78	6.73 ± 2.69	6.44 ± 1.23	6.26 ± 0.24	6.39 ± 0.41
**PUFA**	55.37 ± 6.30	40.17 ± 12.98	45.41 ± 6.99	51.32 ± 1.09	52.78 ± 1.93

Note: Values are presented as the mean ± standard deviation (*n* = 3). Abbreviations: ND: not detected, CH: chloramphenicol, DW: distilled water; AA: ascorbic acid, SA-CDs: *Spatoglossum asperum* carbon dots.

## Data Availability

The original contributions presented in the study are included in the article; further inquiries can be directed to the corresponding author.

## Abbreviations

CDs	Carbon dots
SWCDs	Seaweed-derived carbon dots
HD-CDs	*Halymenia dilatata* carbon dots
GS-CDs	*Gracilaria salicornia* carbon dots
UL-CDs	*Ulva lactuca* carbon dots
CP-CDs	*Caulerpa peltata* carbon dots
SP-CDs	*Sargassum polycystum* carbon dots
SA-CDs	*Spatoglossum asperum* carbon dots
HDP	*Halymenia dilatata* powder
GSP	*Gracilaria salicornia* powder
ULP	*Ulva lactuca* powder
CPP	*Caulerpa peltata* powder
SPP	*Sargassum polycystum* powder
SAP	*Spatoglossum asperum* powder
XPS	X-ray Photoelectron Spectroscopy
FTIR	Fourier-Transform Infrared Spectroscopy
SEM-EDX	Scanning Electron Microscopy–Energy-Dispersive X-Ray spectroscopy
DPPH-RS-A	2,2-diphenyl-1-picrylhydrazyl Radical Scavenging Activity
ABTS-RS-A	2,2-azino-bis(3-ethylbenzothiazoline-6-sulfonic acid) Radical Scavenging Activity
FRA-P	Ferric reducing antioxidant power
MC-A	Metal chelating activity
AP	*Aspergillus parasiticus*
AF	*Aspergillus flavus*
MIC	Minimum inhibitory concentration
MBC	Minimum bactericidal concentration
TSB	Tryptic Soy Broth
MHB	Mueller–Hinton Broth
MTT	3-(4,5-dimethylthiazol-2-yl)-2,5-diphenyltetrazolium bromide
EMEM	Eagle’s Minimum Essential Medium
PV	Peroxide value
TBARS	Thiobarbituric Acid Reactive Substances
CH	Chloramphenicol
AA	Ascorbic acid
DW	Distilled water
PD-PWS	Peeled and deveined Pacific white shrimp
ROS	Reactive oxygen species
EPA	Eicosapentaenoic acid
DHA	Docosahexaenoic acid
PUFA	Polyunsaturated fatty acids
MUFA	Monounsaturated fatty acids
SFA	Saturated fatty acids
PCA	Principal component analysis
